# Biomechanical Characterization of Scallop Shells Exposed to Ocean Acidification and Warming

**DOI:** 10.3389/fbioe.2021.813537

**Published:** 2022-01-20

**Authors:** Aldo Abarca-Ortega, Estefano Muñoz-Moya, Matías Pacheco Alarcón, Claudio M. García-Herrera, Diego J. Celentano, Nelson A. Lagos, Marco A. Lardies

**Affiliations:** ^1^ Departamento de Ingeniería Mecánica, Universidad de Santiago de Chile, Santiago, Chile; ^2^ Center for Biomedical Technology, Universidad Politécnica de Madrid, Madrid, Spain; ^3^ Departamento de Ingeniería Mecánica y Metalúrgica, Pontificia Universidad Católica de Chile, Santiago, Chile; ^4^ Centro de Investigación e Innovación para el Cambio Climático (CiiCC), Facultad de Ciencias, Universidad Santo Tomás, Santiago, Chile; ^5^ Departamento de Ciencias, Facultad de Artes Liberales, Universidad Adolfo Ibañez, Santiago, Chile

**Keywords:** biomechanics, bivalves, elastic anisotropy, mechanical properties, FEA

## Abstract

Increased carbon dioxide levels (CO_2_) in the atmosphere triggered a cascade of physical and chemical changes in the ocean surface. Marine organisms producing carbonate shells are regarded as vulnerable to these physical (warming), and chemical (acidification) changes occurring in the oceans. In the last decade, the aquaculture production of the bivalve scallop *Argopecten purpuratus* (AP) showed declined trends along the Chilean coast. These negative trends have been ascribed to ecophysiological and biomineralization constraints in shell carbonate production. This work experimentally characterizes the biomechanical response of AP scallop shells subjected to climate change scenarios (acidification and warming) *via* quasi-static tensile and bending tests. The experimental results indicate the adaptation of mechanical properties to hostile growth scenarios in terms of temperature and water acidification. In addition, the mechanical response of the AP subjected to control climate conditions was analyzed with finite element simulations including an anisotropic elastic constitutive model for a two-fold purpose: Firstly, to calibrate the material model parameters using the tensile test curves in two mutually perpendicular directions (representative of the mechanical behavior of the material). Secondly, to validate this characterization procedure in predicting the material’s behavior in two mechanical tests.

## 1 Introduction

It is known that about one third of the CO_2_ emissions produced by human activities are deposited on the ocean surface (Sabine et al., 2004). In addition to global warming effects, the CO_2_ sinks through the ocean surface, altering the pH of the seawater and its carbon concentration, a process known as Ocean Acidification (OA) to describe the reduction of the pH level of seawater (Wigley et al., 1996). By the end of this century, seawater is expected to become more acidic, changing its pH from ca. 8.0 to 7.8, and reaching pH 7.6 by the year 2200 (Wolf-Gladrow et al., 1999). There is evidence that the Chilean coastal upwelling areas (30° South latitude) have naturally low pH (acidic) and high concentrations of dissolved carbon dioxide (Torres and Ampuero, 2009; Ramajo et al., 2019; Ramajo et al., 2020; Lagos et al., 2021), which have been projected to be more prolonged events due to the effects of global warming in the following decades (Bakun, 1990; Kim et al., 2013; Wang et al., 2015). These conditions are exposed to progressive acidification described for other coasts such as Oregon and California upwelling ecosystems (Feely et al., 2008; Gruber et al., 2012; Kim et al., 2013). In these regions, the significant impacts of ocean acidification were evidenced in a substantial reduction in the production of the cultured oyster *Crassostrea gigas* (Barton et al., 2012). Due to this evidence and the similarity with the upwelling conditions operating along the Chilean coast, it can be suggested that these environmental impacts can also be affecting the production and sustainability of the scallop aquaculture industry in northern Chile, which have also shown a decreasing trend in landings over the last 2 decades (Lagos et al., 2016; Lardies et al., 2017). A recent study suggests that *Argopecten purpuratus* (*AP*) in its juvenile stage prioritizes calcification at the expense of growth under acidic conditions, being a factor not so sensitive compared to the temperature of its habitat, which is associated with early or late mortalities, observing significantly higher mortality at 18°C than at 14°C (Ramajo et al., 2019; Ramajo et al., 2020).

Over the decades, it has been said that the strength and mechanical properties of the shell in scallops is a function of shell geometry (thickness, height, corrugation, and convexity), and material properties (Currey, 1964; Currey, 1970; Pennington and Currey, 1984; Currey, 1998). The mechanical characterization based on uniaxial tensile tests on mollusk shells has been widely studied during the last decades, being the most predominant of those studies on nacre (Barthelat and Espinosa, 2007; Chen et al., 2017). For example, in 2016, Chen et al. perform a mechanical characterization of shells (Indonesian white pearl oysters) subjected to uniaxial tensile stresses, designing grips and specimens for this purpose, in addition to analyzing the fracture of the material by acoustic emission measurements (Chen et al., 2017). Nevertheless, in the particular case of *AP* (or similar shells of this size), it is the first time that mechanical studies of tensile and flexo-compression tests have been performed and reported. There are very few systematic studies aimed at understanding the impacts of the climate stressors like OA and warming upon the biomechanical properties of the shells. An essential condition to consider due to reports that shell corrosion occurs daily in mussels inhabiting the intertidal zone because they are unable to regulate increases in extracellular *p*CO_2_, which occur at the mantle shell interface during emersion (Melzner et al., 2011). For instance, recent research studied the effect of acidity (increased CO_2_ concentration) on oysters (*Magallana hongkongensis*), concluding a readjustment in the structural integrity, crystallographic orientation, and mechanical characteristics of the shell, constituting a decrease in the stiffness of the material in terms of micro-scale properties (Wolfe et al., 2013; Meng et al., 2019). Other studies show the harmful effects of environmental acidity on marine animals’ structure and molecular composition, affecting their mechanical response; this is how coralline algae (*Lithothamnion glaciale*) are involved in their absorption of nutrients in acidic environments, increasing their structural stiffness (Dery et al., 2017). Mackenzie et al. evaluated the effects of acidification (ambient pH −0.4 pH units) and warming (ambient temperature + 4°C) on the strength of *Mytilus edulis* bivalve shells when fed for a limited period (4–6 h day-1) (Mackenzie et al., 2014). Another research describes an increase in the stiffness of the material of echinoderms (*Eucidaris tribuloides*) when subjected to acid or corrosive environments, postulating a response to predation. Still, it affects the energy allocation of the animal in its growth (Ragazzola et al., 2016). Fitzer et al. evaluated the fracture resistance of *Mytilus edulis* shells under the effects of climate change, in which mollusk was cultured at projected levels of *p*CO_2_ and increased temperatures (Fitzer et al., 2015). Recent research carried out studies on juvenile *AP* (same samples used for the present study) measuring organic content and composition, crystallography and biomechanical properties (to compression), results that indicate significant changes in the composition of the outer part of the shell and also showed that acidification increases the orientation disorder of calcite crystals and reduces their mineral density around 20% (Lagos et al., 2021), which validates what Ramajo et al. study (Ramajo et al., 2019). However, in the groups studied, the only significant differences found in mechanical properties at compression were between test directions, i.e., orthotropic anisotropy was found precluding. Therefore, the possibility of bimodularity or extending the study to other types of tests.

It has been proven that mechanical testing and characterization of materials are paramount in predicting the more complex structures they are composed of. This task is generally done using computational analysis tools, as is the case with the finite element method (FEM). The experimental procedure commonly used to characterize materials is based on standards in its design, use, and measurement (Luxner et al., 2009; Fabris et al., 2019; Stoeckl et al., 2021). However, when the samples have very complex and non-standard geometry, it is necessary to validate the applied methods by computational numerical analysis. In the case of mechanically analyzing a material that presents a complex and unmodifiable geometry, it is imperative to use an adjustment of the properties through numerical simulations, since the standard tests will reflect both the behavior of the material and the influence of the geometry (Bustos et al., 2016). In the particular case of this research, mollusk shells are an extremely fragile material, so manufacturing standard specimens is not possible and the intrinsic geometry of the animal will be a point of study through computational tools. A powerful and highly accurate tool for digitizing real geometries, among other studies, is computational microtomography (micro-CT) scanners, where 3D reconstruction volumes can be obtained layer by layer, and then mesh this geometry in order to perform a numerical simulation using the FEM to evaluate the mechanical response with high accuracy (Arabnejad et al., 2016; Wu et al., 2018; Cheong et al., 2020). Recent studies have used this technology to examine the internal architecture of marine organisms, evaluating porosity, pore size and interconnectivity, and anisotropic features (Martins et al., 2021). The model generated using micro-CT technology allows to perform an inverse adjustment of the mechanical properties in these complex geometries and thus compare the experimental mechanical response of the tests with the corresponding numerical simulation (Bustos et al., 2016).

The aim of this work is the experimental characterization of the anisotropic biomechanical behavior of *AP* shells at the macro level, which have been subjected to different climate change scenarios in its juvenile stage, and thus evaluate if the environmental physicochemical variables, expected in the future due to the influence of climate change, affect the mechanical properties of the shells in the most critical growth stage of the species. Considering these factors, two experimental tests have been designed and proposed—tensile test in two orthogonal directions representative of the material behavior: longitudinal (90°) and transversal (0°), and a tensile test in the diagonal direction (45°); and the flexo-compression test on the complete shell, based on the existing bibliography—implementing an orthotropic linear elastic model for the brittle shell material. The second objective of this research is, once the experimental tests have been performed and considering the complex and non-standard geometries used, to achieve an adjustment in the macro-level biomechanical properties obtained from the experimental tests. The adjustment is performed by numerical simulations using FEM and, through an iterative process, adjust the mechanical properties, first in the longitudinal direction (90°) and then the transversal direction (0°). Finally, the results are validated with numerical simulations of the two proposed tests. The tensile test on the specimen corresponds to the diagonal direction (45°) and the flexo-compression test—using the mechanical properties of the thickness direction from the literature—on the entire shell. Using micro-CT technology, the three specimens and the complete shell are reconstructed.

## 2 Materials and Methods

### 2.1 Sample Collection

Shells of Chilean scallop AP, in its juvenile stage (< 20 mm shell length and without the presence of gonads), were provided by the company INVERTEC S.A. located at Tongoy Bay (30.160 S 71.350 W) from wild populations in 2017, constituting a total of 200 animals. These were transported in a controlled and isolated environment submerged in seawater to the laboratories to experiment. They were fed during the acclimatization period (12 days) following the protocol described by Ramajo et al. (2016). These animals were used for the research reported by Ramajo et al., where the animal’s physiological response to climatic changes is evaluated and for the one described in the present research (Ramajo et al., 2019). Before starting the acidification and temperature treatments on the live animals, 192 scallops were visually checked for organisms that could potentially damage the shells, focusing on *polychaete Polydora sp.* (Radashevsky and Cárdenas, 2004). The four treatments that will be given to the live animals consist of combining temperature and acidification scenarios of the environmental seawater were: 1) 14°C and pH 8.1 present-day averaged conditions observed in Tongoy bay where scallops are cultivated (i.e., control); 2) 14°C and low pH 7.6 resembling acidification condition at control temperature; 3) 18°C and control pH 8.1 representing warming conditions; and 4) 18°C and pH 7.6 representing the combined effect of warming and acidification. Control temperature represents averaged conditions recorded during the scallop collection, whereas high-temperature treatment, in addition to the projected increase in 4°C (IPCC 2014), represents the maximum surface temperature (18°C) reported for Tongoy Bay in the summer season (Lagos et al., 2016). In each scenario, the temperature was stabilized using chillers (± 0.1°C).

The pH scenarios were achieved by bubbling only atmospheric air into an experimental aquarium for present-day control conditions (i.e., < 400 *μ*atm *p*CO_2_ in seawater for 2015) and bubbling a compressed (117 psi) mix of air plus pure CO_2_ and bubbled into the aquarium using mass flow controllers (MFCs, AalborgTM), reaching 900 *μ*atm *p*CO_2_ in seawater, which resulted in a drop of pH 0.3 units, yielding a target pH level of 7.6 for the acidification scenario, while the present-day pH level remained at pH 8.0 units. These *p*CO_2_ levels in seawater take into account the rate of change projected for the atmospheric *p*CO_2_ by 2100, which agrees with the IPCC A2 emission scenario (Meinshausen et al., 2011). The seawater of each aquarium was replaced every 2 days using the pre-equilibrated seawater. The resulting mean (± SE) conditions in temperature, pH, and *p*CO_2_ in seawater for each treatment are previously described (Lagos et al., 2016; Lardies et al., 2017). The experimental exposure lasted 20 days. After the experimental period, the animals were euthanized to make measurements of the shells and tissue weight (Lagos et al., 2016; Lardies et al., 2017), and the shells were dried at 60°C for 4 h and were then stored at ambient temperature and isolated until performing the biomechanical characterization. The shell length used for biomechanical characterization is no longer than 5.5 cm.

In the tensile and flexo-compression tests, the scallop shells were respectively used in a wet condition, considering the same artificial seawater in which the shells were previously immersed (i.e., 33 PSU in salinity). All the material tested was previously reviewed by visual inspection in a microscope. If fractures were found, the shells were discarded since they would not constitute a correct test and subsequent interpretation of the results. At the inspection time, approximately 5% of the total samples showed significant cracks affecting the test results, which were discarded. In the tensile tests, the number of samples depends on the orientation. The minimum number of samples tested per group was two 2) in the 0° orientation in the 8.1 pH group and 18°C (all other groups were a minimum of four 4) samples per group). For flexo-compression tests, five 5) full valves were used for each group, a total of 20 valves. In the Supplementary Appendix S1 a detailed description of the geometries of the specimens used for the mechanical tests carried out in this study can be found.

The Ethics Committees approved all animal care and experimental procedures of the Universidad Santo Tomás and the Universidad de Santiago de Chile (IE N°0146), and they were conducted according to the Guide for the Care and Use of Laboratory Animals published by the US National Institutes of Health (NIH Publication No. 85–23, revised 1996).

### 2.2 Uniaxial Tensile Test

**FIGURE 1:**
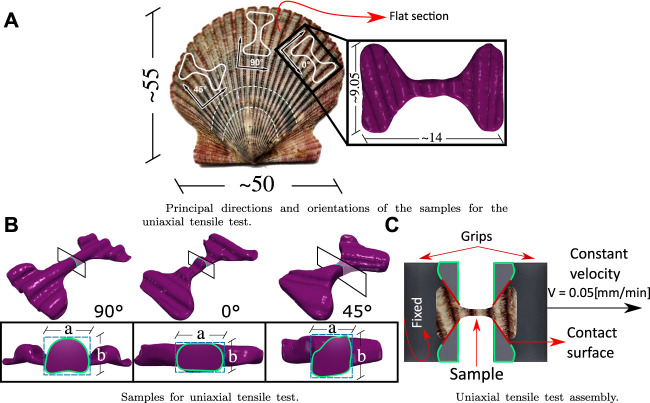
Specification for grips and samples for the tensile tests: **(A)** shows the sample design for the tensile test, the principal’s directions, and orientations of the samples. **(B)** shows the cross-sectional area of the samples and how it is estimated. **(C)** shows the tensile test assembly. All dimensions are shown in mm.

Tensile tests were performed on an Instron 3342 universal testing machine at a grip test speed of 0.05 (mm/min). The fabrication of the samples for the test was based on the geometry of the ASTM E8/E8M standard (ASTM Int, 2016) for flat samples and previous research (Chen et al., 2017), the main focus being on a reduced size and ultimate stress in the center of the sample. The curvature of the shell must also be considered, so the samples were taken from the outermost areas of the shell that present a similar age between specimens. Figure 1 shows the generalities and essential dimensions of the samples for the tensile test. There are three main directions to capture the anisotropy of the material subjected to this stress (90°, 0°, and 45°) which is shown in Figure 1A. The initial transversal area A of each specimen is estimated A = a⋅b as can be seen in Figure 1B, where the dimensions a and b are measured with a micrometer [Mitutoyo ± 0.005 (mm)]. The general set-up of the test is shown in Figure 1C.

A Dremel 3000 2/30 ACC multi-purpose cutting tool was used to manufacture the samples. The flat sections (Figure 1A) were then moved to a laser cutter (80 W nominal power) at low power (20% capacity), cutting the shape of the sample using CAD/CAM technology.

### 2.3 Flexocompression Test

**FIGURE 2:**
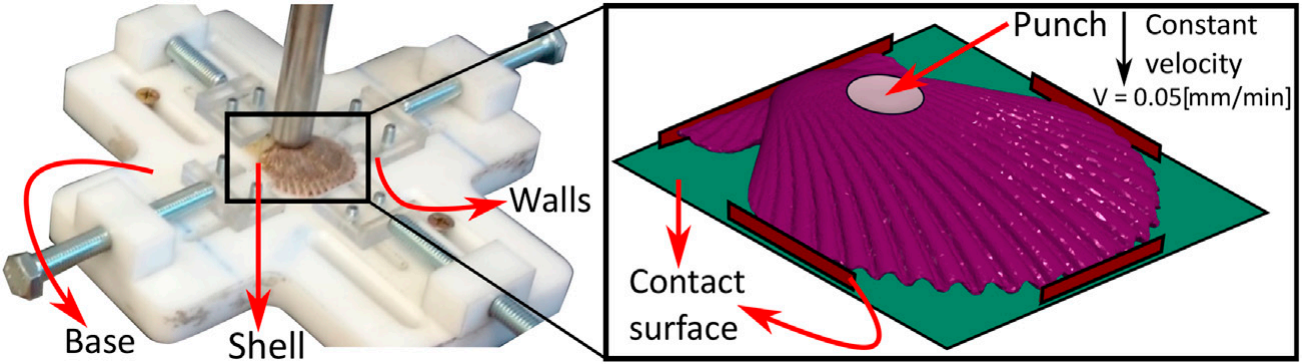
Flexocompression test configuration: The base for the flexo-compression test and the test configuration are shown, in which the punch is located at the cusp of the shell (outer surface).

Flexocompression tests were performed on an Instron 3342 universal testing machine at a punch test speed of 0.05 (mm/min). The flat punch [*∅* 17 (mm)] was positioned at the top of the valve curve. The full valve is positioned on a base with four supports designed and manufactured in Ertacetal (high resistance polycarbonate) and machined with a CNC milling machine (Fanuc Saeil TNV-40). Figure 2 shows the design and configuration of the base for the flexo-compression test.

### 2.4 Statistical Methods

All statistical analyses were carried out employing a one-way analysis of variance (ANOVA). Assumptions of normality and homoscedasticity of the one-way ANOVA were evaluated using the Kolmogorov–Smirnov and Burtlett tests, respectively (R Sokal and Rohlf, 2013). A significance of 0.05 was used to establish significant differences in the biomechanical properties of the individuals across treatments. The ANOVA analysis was followed by a Tukey test when there was the same number of samples in each group or treatment or a Tukey-Kramer test when the number of data between the groups differed.

### 2.5 Constitutive Models

Calcareous materials present viscoelastic and non-linear behavior. However, it is considered by a large number of authors as an anisotropic linear elastic material (Katz and Meunier, 1987). This simplification of its behavior depends on the stress to which it is submitted, where the quasi-static and non-impact process is valid (Velázquez et al., 2015). Thus, the behavior of hard tissues will be governed by the generalized Hooke’s law written as:

$$\sigma =C\cdot \varepsilon \to \left\{\begin{array}{c} \sigma_{1}\\ \sigma_{2}\\ \sigma_{3}\\ \tau_{12}\\ \tau_{23}\\ \tau_{13} \end{array}\right\}=C\cdot \left\{\begin{array}{c} \varepsilon_{1}\\ \varepsilon_{2}\\ \varepsilon_{3}\\ \gamma_{12}\\ \gamma_{23}\\ \gamma_{13} \end{array}\right\}$$ (1)

where *σ* is the tension vector, *C* the stiffness, and *ɛ* the deformation vector. The relationship shown in Eq. 1 is reversible, i.e.:

$$\varepsilon =C^{-1}\cdot \sigma$$ (2)

If the material has two or three orthogonal symmetrical axes, it is an orthotropic material, where its elastic properties depend on the axis in which they are being measured. Following symmetry considerations, the inverse stiffness matrix for an orthotropic material is given by:

$$C^{-1}=\left[\begin{array}{cccccc} \frac{1}{E_{1}} & -\frac{\nu_{12}}{E_{1}} & -\frac{\nu_{13}}{E_{1}} & 0 & 0 & 0\\ -\frac{\nu_{21}}{E_{2}} & \frac{1}{E_{2}} & -\frac{\nu_{23}}{E_{2}} & 0 & 0 & 0\\ -\frac{\nu_{31}}{E_{3}} & -\frac{\nu_{32}}{E_{3}} & \frac{1}{E_{3}} & 0 & 0 & 0\\ 0 & 0 & 0 & \frac{1}{G_{12}} & 0 & 0\\ 0 & 0 & 0 & 0 & \frac{1}{G_{31}} & 0\\ 0 & 0 & 0 & 0 & 0 & \frac{1}{G_{23}} \end{array}\right]$$ (3)

As the stiffness matrix is symmetrical, then there are 12 elastic constants:

• Elastic modulus: *E*_1_, *E*_2_, and *E*_3_.
• Poisson’s ratio: *ν*_12_, *ν*_13_, *ν*_21_, *ν*_23_, *ν*_31_, and *ν*_32_.
• Shear modulus: *G*_12_, *G*_13_, and *G*_23_.

Of these constants only 9 are independent: *E*_1_, *E*_2_, *E*_3_, *ν*_12_, *ν*_13_, *ν*_23_, *G*_12_, *G*_13_, and *G*_23_. These values can be obtained by uniaxial mechanical tests in various directions.

For the estimation of Poisson, 0.2 (Stempflé et al., 2010) will be used in all directions, considering the shell as a non-ferrous material (Blau and Davis, 2000). The shear modulus is estimated for each orientation using the relationship proposed by Huber (Huber, 1923; Panc, 1975), for orthotropic materials in their linear elastic zone:

$$G_{ij}=\frac{\sqrt{E_{i}E_{j}}}{2\left(1+\sqrt{\nu_{ij}\nu_{ji}}\right)}$$ (4)

### 2.6 Numerical Simulations

To perform a numerical fitting of the parameters obtained experimentally by the mechanical tests, numerical simulations are made using the finite element method (FEM). Then the results are analyzed following the same procedures and experimental methodologies described above. One of the problems described by several authors is the complex geometry of the shells, so it was decided to use microCT scans with high enough accuracy (Bruker Skyscan 1278) to acquire the essential structural details. Thus, full-shell geometries and tensile specimens in the three different orientations were obtained with an accuracy of 50 microns. The scanned specimens are of the same age and growth groups as the experimental samples. All simulations were carried out using VULCAN, an in-house FEA software. Each mesh was composed of linear tetrahedral elements due to the complexity of the scanned geometries, ensuring that the integrity of the details is maintained with the least possible alteration. Therefore, the number of elements of the simulations is of the order of two hundred thousand elements for the uniaxial tensile test simulations and five hundred thousand elements for the flexo-compression simulations, ensuring a minimum dihedral angle of 20.8°.

#### 2.6.1 Coordinated System of Elements

Considering the shell as a material of orthotropic behavior (Katz and Meunier, 1987), and due to the complex geometries present in the experimental tests carried out, an adjustment was made to the coordinate system of each element of the mesh as a function of the orthotropy. A numerical method was developed capable of representing the shell geometry by adjusting parametric surfaces to the barycenters of the tetrahedral elements that compose the mesh, thus calculating the three directions of the orthotropic behavior of the geometry.

**FIGURE 3:**
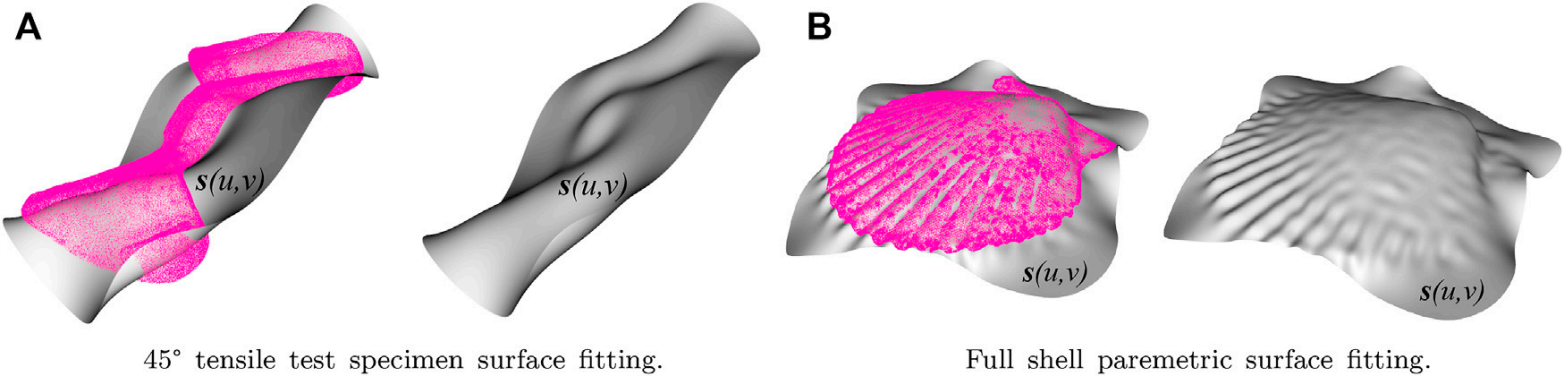
Parametric surfaces adjusted to the barycentres of meshes for 45° tensile and whole shell specimens: **(A)** shows the 45° tensile test specimen surface fitting, and **(B)** shows the entire shell parametric surface fitting.

The process required meshing the solid, either the shell or the tensile specimen, and the barycenters of each of the tetrahedral elements that make up the mesh were calculated. Once coordinates were obtained, the library “The Point Cloud Library (PCL)” (Rusu and Cousins, 2011), an open-source library of algorithms for point cloud processing and 3D geometry processing tasks, was used. That algorithm was modified so that the barycenters represent the point cloud and run the B-spline fitting on this point cloud to obtain a smooth, parametric surface. Assuming that a surface has a parametric form Γ: ***s*** (*u*, *v*) in $R^{3}$ , the program editing consisted of selecting the parameters for the B-spline surface fitting (the surface refinement and the number of iterations) and visualizing each refinement and iteration step until the surface was adapted to the point cloud, as can be seen in Figure 3.

**FIGURE 4:**
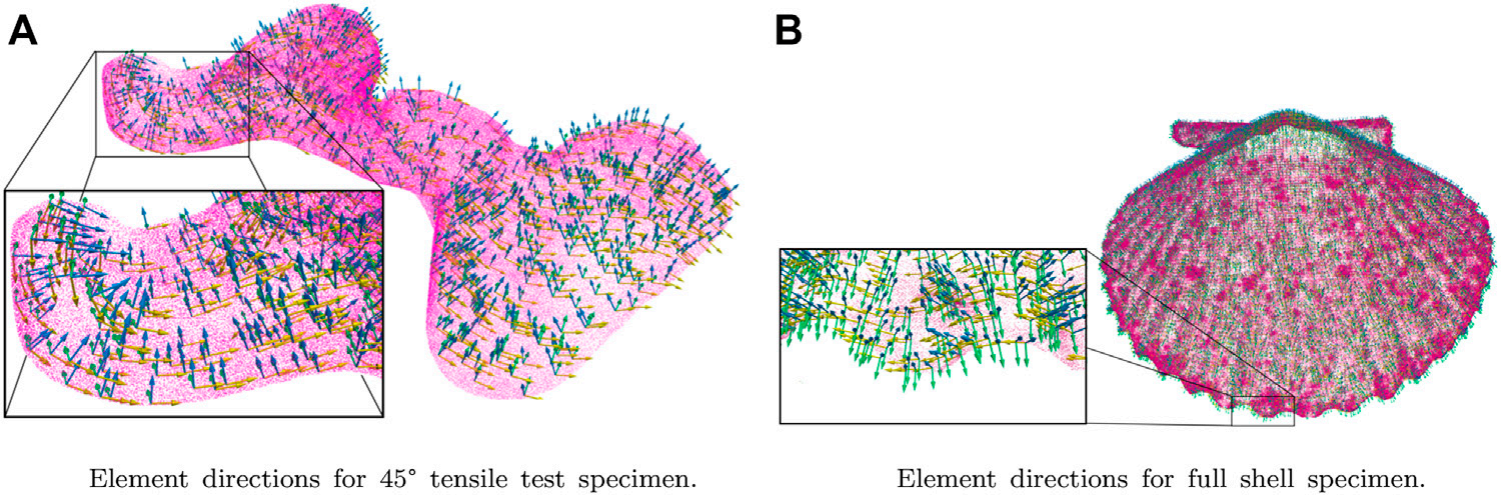
Element directions fitting for meshes used in FEM simulations: The boxes are shown the orientations of the particular coordinated system concerning a global orthotropic behavior. **(A)** shows the element directions for the 45° tensile test specimen, and **(B)** shows the element directions for the entire shell specimen.

Once the parametric surfaces were fitted to the mesh, the direction normal to the surface, the first of the three orthogonal directions, was calculated. This procedure was performed by orthogonally projecting the barycenter of each element to the parametric surfaces representing the geometry. Li et al. (2019) developed an orthogonal projection algorithm whose tremendous computational speed was used for this procedure due to the abundant mesh elements. This code calculates the minimum distance between a point and a parametric surface and then returns the coordinates to each barycenter projection (footpoint). Subsequently, vectors were created from the footpoint to its corresponding barycenter, representing the direction normal to the surface. The other two directions were obtained by creating a vector from the origin of the shell ribs to each of the mesh barycenters. Finally, cross products were performed to calculate the longitudinal and transversal directions, as shown in Figure 4.

#### 2.6.2 Uniaxial Tensile Test

The tensile test simulation considers the three specimen geometries according to their direction (90, 0, and 45°), which will behave hypothetically following an orthotropic linear elastic model. The calculation of the cross-sectional area is the same followed experimentally, described in Figure 1B. The specimen grip will act as rigid surfaces on the specimen, and the contact model used [a complete description of the contact penalty method is described by Cañas et al. (2018)] is considers that the contact pressure [*p*_*n*_ (MPa)] increases quadratically with the penetration [*g*_*n*_ (mm)] of the surfaces, i.e.:

$$P_{n}=E_{n0}g_{n}+E_{n}g_{n}^{2}$$ (5)

Where *E*_*n*0_ (N/mm^3^) and *E*_*n*_ (N/mm^4^) are constants that control the penetration. Due to the high number of simulations, a code was developed to adjust these constants to avoid excessive penetrations erroneous results automatically. These simulations will be used to adjust the elastic parameters numerically, as explained below.

#### 2.6.3 Flexocompression Test

The numerical simulation of this test, which will validate the tests and adjusted results, has the complete shell with a linear elastic orthotropic material and several surfaces that will contact the specimen. These contacts respond to the different planes that interact with the specimen, restricting its displacement or applying force, as shown experimentally in Figure 2. The contact model used is shown in Eq. 5.

#### 2.6.4 Fitting Procedure

**FIGURE 5:**
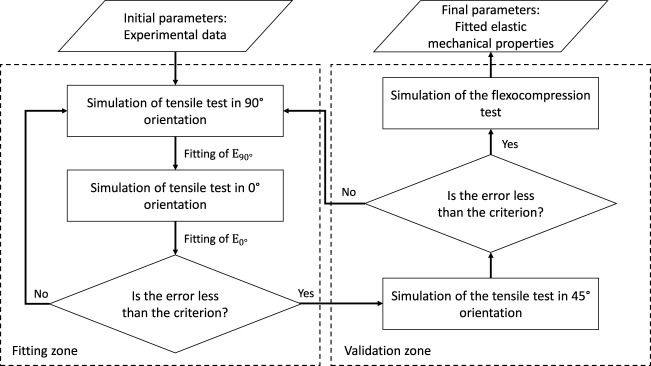
Flowchart of the fitting procedure of elastic properties through numerical simulations.

Having adopted the orthotropic linear elastic model and considering many parameters, a proper fit strategy must be adopted through numerical simulations. This is how, and according to Figure 5, we follow an iterative process until a minimum error criterion is satisfied concerning the experimental data.

Figure 5 represents how the numerical fitting of the properties obtained experimentally will be the input data for the numerical simulations, and the following steps will be done until a satisfactory adjustment is achieved.

Step 0 With the experimental properties and considering an orthotropic behavior, they are coupled to the tensile models and started simulations. First, the shear modulus will be calculated according to Huber’s equation (Huber, 1923), as explained above.

Step 1 Tensile simulations are run at 90° and 0° to obtain the resulting force on a pair of cooperative grip contact surfaces. The same experimental methodology will be followed to obtain the specimen’s behavior in strain versus stress, and thus the elastic modulus will be adjusted concerning the initial experimental one. This step is repeated until the error criteria are satisfied, explained below.

Step 2 Once the error criteria for the 90° and 0° simulations have been satisfied, these results are validated by performing the 45° tensile simulation. If the error exceeds the chosen criterion, the fittings performed will be reanalyzed. If the error is lower (compared to the experimental results), the process of adjusting the model from the perpendicular plane stiffnesses has been validated.

Step 3 With the elastic properties adjusted numerically, the simulation of the flexo-compression test is carried out, where the shell will be engaged in the area at the cusp of the shell by a cylindrical flat punch, with a geometry similar to that used experimentally. Finally, the results are compared with those obtained in the tests to observe the behavior of the material’s final adjusted mechanical properties to validate the mechanical tests.

The error for each step of data fitting in the numerical simulation is quantified through the normalized root-mean-square deviation (NRMSD) to obtain a comparable value with the experimental curve. This parameter is defined by Eq. 6.

$$\text{NRMSD}=\frac{1}{\Delta }\sqrt{\frac{1}{n}\overset{n}{\underset{i=1}{\sum }}{\left(y_{i}-\hat{y_{i}}\right)}^{2}}$$ (6)

where *n* is the number of experimental data, *y*_*i*_ are the experimental measures, $\hat{y_{i}}$ is the adjusted numerical value, and Δ is defined as shown in the Eq. 7.

$$\Delta =|y_{\max}-y_{\min}|$$ (7)

To obtain the NRMSD in this particular case, the values to be considered in *y* is the measure of engineering stress, both experimental and numerical, at the same point of engineering strain at each spot. The adjustment of the mechanical properties will be made until the NRMSD, our criterion error, are less than 8%. The magnitude of this criterion was chosen considering possible geometrical problems resulting from different sizes with the experiments, which could generate divergences in the adjustments with smaller criteria.

## 3 Results and Discussion

### 3.1 Experimental Tests

#### 3.1.1 Uniaxial Tensile Test

The tensile test described how the sample is placed in the grips, shown as a little curve at the beginning of the force versus displacement curve. Then, for the analysis of the mechanical behavior, the linear elastic section is considered, i.e., where there is real load in the tensile sample, after settlement in the grips, being a linear section up to fracture. Finally, the bending stresses are applied to the samples due to the small curvatures along their length, which is one reason why a numerical fitting of these results should be made as they are not entirely standard test pieces.

**FIGURE 6:**
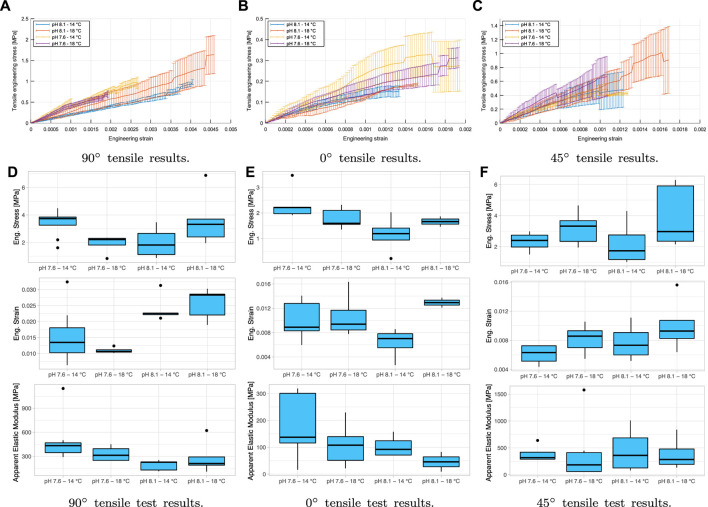
Results of the tensile test apparent behavior in engineering stress against engineering strain: **(A–C)** shows the tensile results for 90°, 0°, and 45° orientations, respectively. **(D–F)** compare the mechanical behavior on the maximum strain, maximum engineering stress, and the elastic modulus. (●): extreme values of the distribution.

Once the force versus displacement curve data has been split, the engineering stress (*σ*) versus engineering strain (*ɛ*) curves will be calculated from the initial dimensions of each sample and the distance between grips at the start of the test. A linear fitting will be made in each experimental test using the least-squares method, thus obtaining each test’s apparent elastic modulus (*E*). Therefore, the ultimate stresses and deformations and the apparent elastic modulus for each group analyzed will be obtained. The results are shown in Figure 6. The results are described as apparent based on the calculation of the area and the complex geometry of each specimen, as can be seen in Figure 1B, which would not directly result in stress, strain, and elastic modulus, leading directly to evaluate and correct the results using numerical simulations with the adopted orthotropic model. Notwithstanding the above, statistical analysis to the uncorrected values to evaluate differences between groups would not be incorrect to perform the adjustment of elastic parameters is considered similar between all of them, as the geometry between specimens is similar.

The mechanical tensile behavior of all groups varies with their orientation. However, it should be noted that the analyses that can be made based on the experimental results do not take into account the particular differences in the geometry of the specimens, which will inherently generate stress concentrations and varying elastic properties. Therefore, the experimental results will be treated as apparent, being the basis for an inverse adjustment of these variables through numerical simulations shown in the following sections. The results of stress, strain and apparent elastic modulus of the uniaxial tensile and flexocompression tests, as well as the extension of the results of the statistical analyses performed can be found in the Supplementary Appendix S2. Nevertheless, the longitudinal (90°) and transverse (0°) orientations are the ones that present the most significant differences, the response to 0° being much weaker. In addition, for all directions, a linear response is observed with the deformation. Thus, this material’s anisotropic and linear mechanical response is consistent with the preferential orientation of the crystals (Taylor and Layman, 1972). After statistically analyzing the tensile test results, it is seen that for the transverse orientation (0°), there are no significant differences between variables measured at all, considering variances and means.

These results are different from those reported previously in the compression behavior where it shows significant differences (*p* < 0.05) in the elastic modulus between the pH 7.6 and 14°C group and all the others, including the control, wet condition, and compression in the thickness (Lagos et al., 2021). The above indicates anisotropic behavior in compression with significantly different properties from each other. Even if there are no significant differences between directions in the tensile tests, they will be considered independent elastic properties for the orthotropic model. Not having significant differences between groups both in stiffness, as in strains or maximum stresses, indicates that the animal adapts to different scenarios of growth to which it is subjected, at least in its juvenile stage before reaching maturity. This adaptation responds to an increase in its metabolism and change in the crystalline composition to maintain mechanical properties in hostile environments, as Ramajo et al. (2019) and Lardies et al. (2017) wrote in their researches. These findings support the hypothesis of biomineralization plasticity as a potentially advantageous compensatory mechanism (Checa et al., 2016a; Telesca et al., 2019).

#### 3.1.2 Flexocompression Test

**FIGURE 7:**
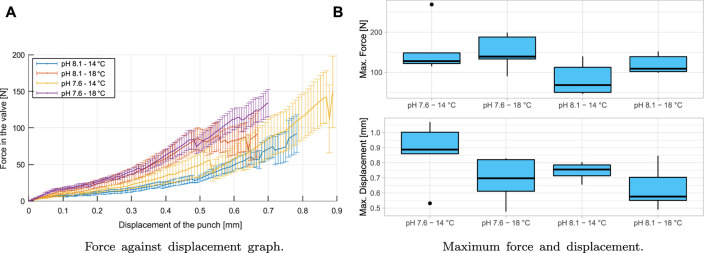
Flexocompression test results: **(A)** shows the behavior of complete shells in a flexo-compression test. It is shown at the application of force on the valve and displacement of the punch (vertical) at the cusp of the shell. **(B)** shows the comparison of the behavior of shells subjected to flexo-compression tests. The top graph shows the maximum force applied to the shell (N), and the bottom graph shows the displacement of the punch (mm) before the fracture. (●): extreme values of the distribution.

Figure 7A shows a representative force curve in the shell against punch displacement when it is in contact with the valve, as can be seen in Figure 2. The four groups up to the fracture are shown, and a non-linear behavior is seen at this forced state.

The minor breaks seen in Figure 7A are because there are cracks in the material, but these are not catastrophic at the end of the test, i.e., the material can withstand the cracks produced. If the cracks have occurred under natural conditions (pH 8.1/14°C), the animal can recover the damaged material if it has adequate food and if the crack is not big enough for the predator to have access to the mollusk’s muscle (Checa et al., 2016b). The material has a brittle behavior, where when passing the elastic zone, a sudden break is produced, representing the critical fracture of the shell. The maximum punch displacement and maximum shell force are shown in Table 1, and it can be seen in the boxplot in Figure 7B.

**TABLE 1:** Maximum values for the behavior of shells in the flexo-compression test. Mean and standard error of force and displacement.

Condition	Force on shell (N)	Displacement of the punch (mm)
pH 8.1–14°C	83.31 ± 18.32	0.74 ± 0.03
pH 8.1–18°C	120.20 ± 10.71	0.63 ± 0.06
pH 7.6–14°C	156.45 ± 28.67	0.87 ± 0.09
pH 7.6–18°C	149.90 ± 19.69	0.69 ± 0.07

According to the analysis of variance, there are no significant differences between groups for variables of forces and displacement (*p* > 0.05). Therefore, the variation of pH and temperature applied to the group and control does not produce significant differences for the flexo-compression test in the variables of displacement and force. These results align with what was described above, where the animal can adapt to changes in its juvenile stage to achieve mechanical properties that are not unfavorable to predation. Therefore, a compensatory mechanism, such as biomineralogical plasticity, maybe working under OA conditions (Leung et al., 2017; Telesca et al., 2019).

### 3.2 Numerical Simulations

Due to the diverse and complex geometries of the specimens studied in the uniaxial tensile and flexo-compression mechanical tests, a correct adjustment of the elastic properties must be made using numerical simulations, i.e., from the apparent properties obtained experimentally, iterate using the finite element method to simulate the experimental tests until similar experimental and numerical results are obtained. The results of the experimental tests obtained indicate that there are no significant differences between groups for their tensile apparent elastic properties. Therefore, the numerical simulations will focus on the control group (pH 8.1 and 14°C) representing the expected behavior. On the other hand, it is interesting to compare what was obtained in tensile tests with the compression properties (Lagos et al., 2021), as, in magnitude, there is a difference close to 82% (based on a relative error centered on the compression properties), which could give signs of bimodular behavior, so, focused on the fitting process in the tensile test, this factor will be evaluated.

#### 3.2.1 Uniaxial Tensile Test

**FIGURE 8:**
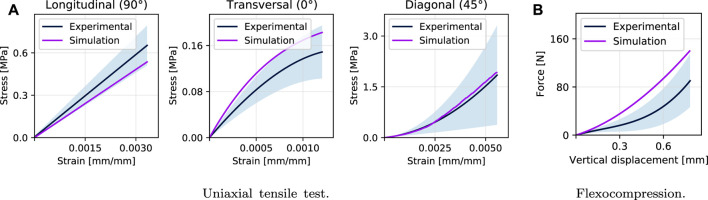
Results of simulations and comparison with experimental tests: **(A)** shows the simulation results of the tensile test, both for the elastic properties fitting stage (longitudinal and transversal direction) and for the validation stage (diagonal). **(B)** shows the validation of the orthotropic model adopted with the adjusted properties obtaining an error of approximately 23%.

Using the orthotropic model described above, the experimental tensile tests are simulated based on the results obtained, using as input variables the Elastic Modulus (E.M. in the following tables) in the longitudinal (90°) and transversal (0°) directions. In contrast, for the direction in the thickness, it is used what is previously reported (Lagos et al., 2021), to compression, for the same group (*E*_*z*_ = 131.28 MPa). After a series of iterations following the process described in Figure 5, an expected error criterion of less than 8% was achieved, as shown in Figure 8 for the simulations to tensile in its longitudinal and transversal directions. Finally, the validation is carried out with the simulation in a diagonal direction (45°), obtaining a minimal error to the experimental curve (2%) having only fitted the elastic properties in the plane. Table 2 shows the results obtained compared to the unfitted ones and shows the NRSMD for each direction.

**TABLE 2:** The table shows the results of the unfitted experimental elastic properties and the fitted elastic properties obtained from the tensile test and their respective fit.

Direction	Initial E.M (MPa)	Final E.M (MPa)	NRMSD (%)
Longitudinal (90°)	199.156	407.947	2.59
Transversal (0°)	57.524	143.633	7.78

Considering the results obtained in the fitting process of elastic properties and comparison to those previously reported in compression tests and the exact orthotropic directions of our tensile tests (Lagos et al., 2021), it is that, although after adjusting the elastic modulus in the Longitudinal (90°) and Transverse (0°) directions, these increase considerably their stiffness, they do not become sufficiently close to the compression properties, reaching relative differences close to 62.5% in the Longitudinal case, as is shown in Table 3. Therefore, it can be said that if a shell is to be wholly known and characterized by these characteristics, the properties of both compression and tension must be known, and a comparative study carried out between them.

**TABLE 3:** The table shows the results compared to the properties previously reported of uniaxial compression tests in the three orthogonal directions compared to those obtained from the tensile test, both experimental and its fitting, and the difference obtained between both calculated as percentage relative error.

Direction	Compression E.M (MPa)	Final E.M (MPa)	Diff
Longitudinal (90°)	1080.287	407.947	62.2%
Transversal (0°)	226.362	143.633	36.5%
Thickness	131.277 (Ramajo et al., 2019)	—	—

#### 3.2.2 Flexocompression Test

After validating the tensile test simulation in the diagonal direction (45°), the numerical simulation of the flexo-compression test is carried out. The same set-up of the experimental test is used (Figure 2), in which the punch, base, and walls were implemented employing quadratic contact elements; the shell was meshed using tetrahedral elements. The mechanical properties obtained by fitting the simulations of the tensile tests were those implemented for this case in a unimodular orthotropic model, according to the process described in Figure 5. In comparison with the experimental results of the flexo-compression test, the results of the simulation are shown in Figure 8B, where a similar behavior can be observed between both studies, obtaining a quantifiable error of 23.33%.

**FIGURE 9:**
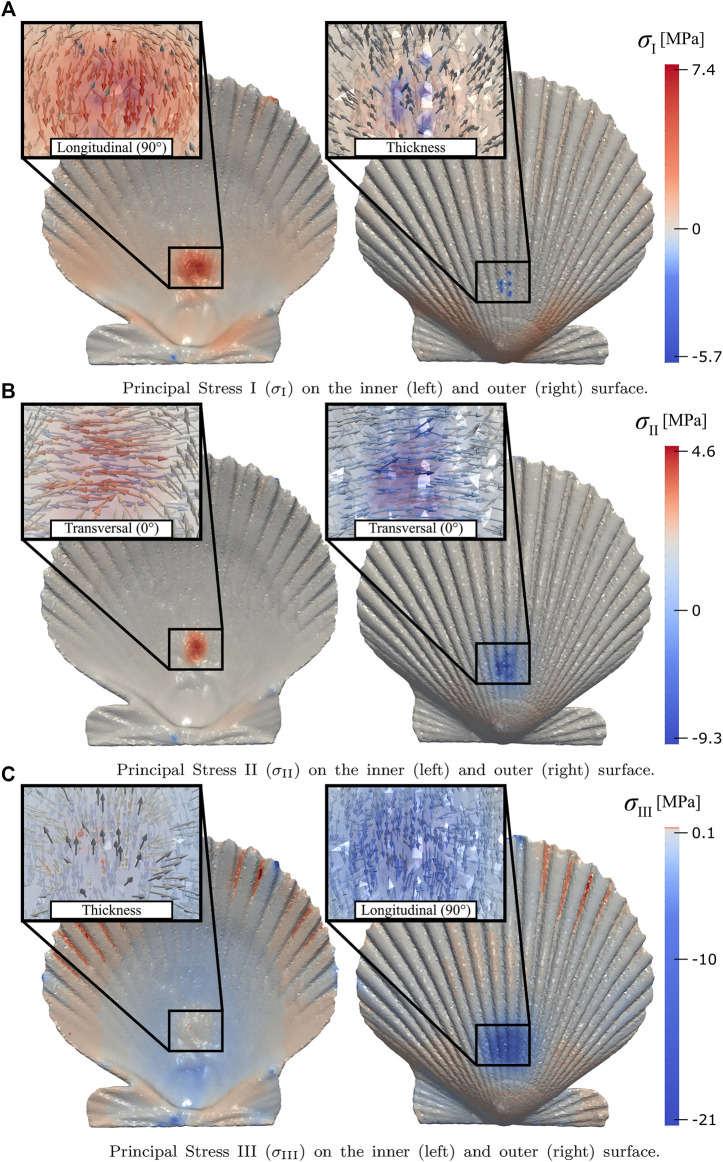
Principal stresses magnitude and directions (in the boxes) in the shell-punch contact area: **(A–C)** show the principal *σ*_I_, *σ*_II_, and *σ*_III_ on the inner (left) and outer (right) surface, respectively.

The simulation analysis is performed when the reaction force is approximately 140 [N], i.e., when the displacement of the simulation and the experimental test coincide. The analysis is focused on the stress concentration in the area where the punch contacts the valve; by mapping the principal stresses *σ*_I_, *σ*_II_, and *σ*_III_ in the shell-punch contact zone (Figure 9). An interesting effect is observed, an approximate alignment between the directions of the principal stresses and the three orthotropic directions of the material, which occurs both on the outer (compression) and inner (tension) surface of the shell. The corresponding alignments for the outer and inner surfaces are shown in Table 4.

**TABLE 4:** Alignment between principal stresses directions (*σ*_I_, *σ*_II_, and *σ*_III_) and orthotropic directions (Longitudinal, Transversal, and Thickness) in the shell-punch contact area produced by the effects of compressive stresses (outer surface) and tensile stresses (inner surface).

Zone	Principal stresses *σ*_I_ *σ*_II_ *σ*_III_		
Outer (Compression)	*σ*_I_ → Thickness	*σ*_II_ → Transversal	*σ*_III_ → Longitudinal
Inner (Tension)	*σ*_I_ → Longitudinal	*σ*_II_ → Transversal	*σ*_III_ → Thickness

The highest magnitudes of tensile and compressive stress in the shell-punch contact zone were distributed in the longitudinal direction, i.e., in the directions parallel to the ribs, which have higher inertia than the rest of the shell. In addition, the compressive stress exceeds the tensile stress, which could indicate that the shell must withstand compressive stress to ensure the survival of the mollusk during local bending caused by predator attacks, which has to be verified with future studies related to this phenomenon.

## 4 Summary and Conclusion

This research was devoted to the characterization of the mechanical behavior of scallops shells subjected experimentally to climate change scenarios for the ocean (acidification and warming). Special devices were designed and manufactured to quantify the mechanical properties of the shell material in quasi-static tests. The methods developed and explained in the present work can be further applied to different species to characterize their biomechanical properties. In addition, the effect on the mechanical behavior of shells during growth conditions under climate change scenarios has been studied.

The results obtained for the uniaxial tensile and flexo-compression mechanical tests do not indicate significant differences between groups under climatic changes, which means that the mechanical properties are not affected by these conditions. However, when the material is subjected to compression, it is affected mechanically, obtaining greater strength and stiffness under more extreme conditions (high temperature and acidity) (Lagos et al., 2021). Temperature and pH alterations lead to different levels of calcification that might be related to a biomechanical adaptation process due to climate change scenarios, a process in line with mineralogical plasticity as a compensatory mechanism to the impacts of ocean acidification (Leung et al., 2017; Telesca et al., 2019). According to the orientation at which they were performed, mechanical anisotropies were found, similar to previous studies (Lagos et al., 2021). After performing tensile tests and their respective parameter fittings and validation, it was found that the shell behaves according to an orthotropic unimodular model. However, according to the results shown in the present study, if one wants to know in depth the macromechanical behavior of this class of bivalves, it is imperative to study the behavior in compression and tension. Scallop shells (*AP*), as they become stiffer and more resistant under acidity conditions and high temperature, indicate some adaptation to extreme controlled environments. Suppose the condition of high acidity and temperature occurs in a natural environment; in that case, the scallop food (protozoa, larvae of other organisms, and algae) will also be affected and reduced, so its chances of surviving without being in artificial hatcheries are significantly reduced. Other factors related to climate change need to be evaluated in further studies.

Therefore, and considering the results obtained in the present study, it can be said that for *AP* it is validly mechanically modeled with a unimodular orthotropic model, where the directions of the principal stresses to which it is subjected are directly related according to its crystalline microstructure (Acevedo et al., 2010; Tippmann and Benavente, 2014; Almagro et al., 2016; Lagos et al., 2021). This is significant due to the survival mechanisms that the animal can develop under mechanical loads, allowing us to know the direction and orientation in which the shell concentrates the principal stresses. Similarly, with this knowledge, it is possible to develop tools to avoid the concentration of stresses in those directions in industrial processes to obtain less material loss in the harvesting stage of the mollusk.

## Data Availability

The raw data supporting the conclusion of this article will be made available by the authors, without undue reservation.
