# Supplementary material for: Biomechanical Characterization of Scallop Shells Exposed to Ocean Acidification and Warming
Source: Front Bioeng Biotechnol. 2022 Jan 20;9:813537. doi: 10.3389/fbioe.2021.813537 (PMC8811142; doi:10.3389/fbioe.2021.813537)
Supplement: Supplementary file 1 [file DataSheet2.pdf]

S2 Appendix. Mechanical test results and statistical analysis

This appendix shows the procedure and statistical outputs of the analyses applied to the results obtained from the experimental mechanical tests of each group studied. All statistical analyses are carried out by means of an analysis of the variance of a route or with a factor (ANOVA), which allows us to contrast the null hypothesis that the averages of the groups (in each factor to be measured) are equal using the Kolmogorov–Smirnov and Burtlett tests, respectively [1]. In order to perform the ANOVA test, it is necessary to make certain assumptions:

1. The independent variable must be measured at least at interval level.
2. Independence of observations.
3. The distribution of the residuals must be normal.
4. Homogeneity of the variances (homocedasticity).

To evaluate the homogeneity of the variances, a Levene test is carried out; it consists in evaluating the equality of the variances of one variable for two or more groups. If the result value of the test is less than a certain level of significance, in this case 0.05, then there are significant differences between the variances of the variable in each group and ANOVA cannot be applied. The analysis of variance evaluates two or more groups, based on the level of significance of the test, if the result is less than 0.05 then there are significant differences between groups. If there are significant differences, the Tukey-Kramer test is performed, which makes multiple comparisons between means of groups once the null hypothesis of equality of means is rejected. The results of the statistical analyses performed are shown in table format, highlighting in bold type those where significant differences exist [2].

1 Flexocompression Test

Table 1 shows the maximum values for the behaviour of valves under the flexocompression test.

Condition	Force in valve [N]	Displacement of the punch [mm]
pH 8.1 - 14 °C	83.31 ± 18.32	0.74 ± 0.03
pH 8.1 - 18 °C	120.20 ± 10.71	0.63 ± 0.06
pH 7.6 - 14 °C	156.45 ± 28.67	0.87 ± 0.09
pH 7.6 - 18 °C	149.90 ± 19.69	0.69 ± 0.07

Table 1: Maximum values for the behaviour of shells in the flexocompression test. Mean and standard error of force and displacement.

Table 2 shows the Levene test for the force and displacement variables in all groups, performed the variance homogeneity test on the data. The result shown in column p (degree of significance) indicates that there are no significant differences between variances, as this is always greater than 0.05. An ANOVA is carried out, as shown in table 3.

According to the analysis of variance shown in table 3 there are no significant differences between groups for force and displacement variables. Therefore, the variation of pH and temperature applied to the control group does not produce significant differences, for the flexocompression test in the displacement and force variables, between the different groups including the control.

	Mean quadratic error	F - test	p
Force	571.803	0.895	0.465
Displacement	0.007	1.087	0.383

Table 2: Levene test for independent variables in flexocompression test.

	Degrees of freedom	F - test	p
Force	3	2.687	0.081
Displacement	3	2.314	0.115

Table 3: Analysis of variance for independent variables in the flexocompression test.

2 Tensile Test

Table 4 shows the results of strain and engineering stress as well as the elastic modulus of each group in the format of mean and standard error for each orientation and humidity state.

State	Group	Orientation	Strain	Stress [MPa]	Elastic Modulus [MPa]
Wet	pH 7.6 - 14 °C	0°	0.002 ±0.000	0.325 ± 0.093	177.97 ± 57.701
		45°	0.001± 0.000	0.497 ± 0.072	390.52 ± 83.669
		90°	0.003 ± 0.000	1.070 ± 0.131	490.06 ± 97.496
	pH 7.6 - 18 °C	0°	0.002 ± 0.000	0.249 ± 0.061	108.27 ± 31.286
		45°	0.001 ± 0.000	0.712 ± 0.208	417.23 ± 241.22
		90°	0.002 ± 0.000	0.641 ± 0.080	331.50 ± 49.756
	pH 8.1 - 14 °C	0°	0.001 ± 0.000	0.134 ± 0.033	103.13 ± 20.748
		45°	0.002 ± 0.000	0.530 ± 0.207	453.06 ± 216.50
		90°	0.004 ± 0.000	0.806 ± 0.147	191.09 ± 28.555
	pH 8.1 - 18 °C	0°	0.002 ± 0.001	0.201 ± 0.023	45.944 ± 36.706
		45°	0.002 ± 0.000	0.799 ± 0.284	385.85 ± 127.44
		90°	0.005 ± 0.000	1.258 ± 0.406	284.86 ± 89.194

Table 4: Uniaxial tensile behaviour data depending on orientation, showing strain and engineering stress and elastic modulus.

Table 5 shows the ANOVA test for the elastic modulus in the uniaxial tensile test results. According to the results in columns *p-value*, there are no significant differences between any group.

Orientation	Group	*p-value*			
90°	8.1pH-14°C	x			
	8.1pH-18°C	0.883	x		
	7.6pH-14°C	0.127	0.402	x	
	7.6pH-18°C	0.762	0.988	0.690	x
45°	8.1pH-14°C	x			
	8.1pH-18°C	0.996	x		
	7.6pH-14°C	0.997	1.000	x	
	7.6pH-18°C	0.999	0.999	1.000	x
0°	8.1pH-14°C	x			
	8.1pH-18°C	0.917	x		
	7.6pH-14°C	0.647	0.478	x	
	7.6pH-18°C	1.000	0.896	0.618	x

Table 5: Analysis of variance for the elastic modulus in the uniaxial tensile test at various orientations.

References

[1] R. R. Sokal and F. Rohlf, "Biometry : the principles and practice of statistics in biological research / robert r. sokal and f. james rohlf," *SERBIULA (sistema Librum 2.0)*, 04 2013.

[2] M. B. Brown and A. B. Forsythe, "Robust tests for the equality of variances," *Journal of the American Statistical Association*, vol. 69, no. 346, pp. 364–367, 1974.
