# Supplementary material for: Biomechanical Characterization of Scallop Shells Exposed to Ocean Acidification and Warming
Source: Front Bioeng Biotechnol. 2022 Jan 20;9:813537. doi: 10.3389/fbioe.2021.813537 (PMC8811142; doi:10.3389/fbioe.2021.813537)
Supplement: Supplementary file 2 [file DataSheet1.pdf]

S1 Appendix. Specimen geometries and dimensions

This appendix shows the geometries and dimensions of specimens to be considered for each test performed. The magnitudes shown define the mean ± standard error of each dimension in millimetres.

1 Flexocompression Test

Figure 1 shows the geometry of the entire valve and the dimensions to be considered. Table 1 shows the dimensions for each group (in millimetres), showing its interior (i) and exterior (e).

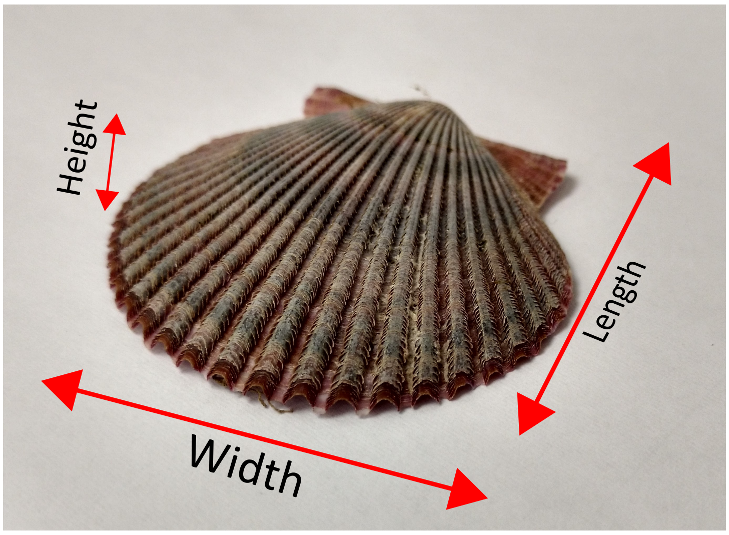

Figure 1: Geometric dimensions of complete valve, indicating its height, length and width.

Group	Length (i)	Length (e)	Width (i)	Width (e)	Height (i)	Height (e)
pH 8.1 - 14°C	41.13 ± 0.45	43.29 ± 0.27	41.48 ± 0.29	44.60 ± 0.29	3.27 ± 0.08	3.80 ± 0.17
pH 8.1 - 18°C	42.01 ± 0.31	44.41 ± 0.37	41.15 ± 0.33	45.80 ± 0.46	3.06 ± 0.21	3.44 ± 0.24
pH 7.6 - 18°C	41.07 ± 0.72	43.29 ± 0.71	41.12 ± 0.46	45.00 ± 0.86	3.24 ± 0.14	3.30 ± 0.24
pH 7.6 - 14°C	41.53 ± 0.82	42.55 ± 0.85	40.43 ± 0.39	42.65 ± 0.99	3.41 ± 0.20	3.92 ± 0.28

Table 1: Geometric data of flexocompression specimens, showing means and standard errors. All dimensions shown are in millimeters. The letter *i* denotes *inside* and *e* denotes *exterior*.

2 Uniaxial Tensile Test

Figure 2 shows the uniaxial tensile test specimens manufactured from small flat pieces of the valve on the outside and cut in laser machine in the dog bone shape, also indicating their geometric dimensions. Table 2 shows the geometric dimensions of the tested specimens in millimetres.

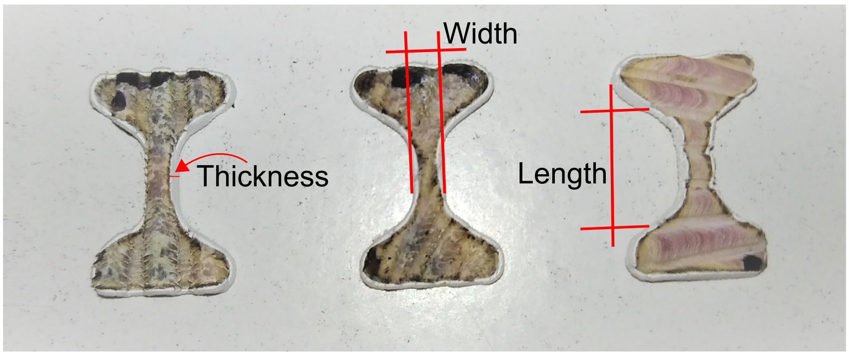

Figure 2: Specimens at 90°, 45°, and 0°, respectively, manufactured on a laser cutter at low power. The figure indicates the geometric dimensions of the specimen.

State	Group	Orientation	Width [mm]	Length [mm]	Thickness
Dry	pH 7.6 - 14 °C	0°	1.79 ± 0.11	5.58 ± 0.17	0.97 ± 0.10
		45°	1.75 ± 0.08	5.50 ± 0.13	1.48 ± 0.12
		90°	1.90 ± 0.06	5.75 ± 0.09	1.21 ± 0.07
	pH 7.6 - 18 °C	0°	1.89 ± 0.05	5.73 ± 0.08	0.94 ± 0.08
		45°	1.92 ± 0.02	5.78 ± 0.04	0.98 ± 0.09
		90°	1.75 ± 0.06	5.51 ± 0.10	1.01 ± 0.08
	pH 8.1 - 14 °C	0°	1.27 ± 0.06	5.86 ± 0.09	1.98 ± 0.12
		45°	1.69 ± 0.08	5.42 ± 0.12	1.33 ± 0.07
		90°	2.05 ± 0.09	5.02 ± 0.14	2.11 ± 0.15
	pH 8.1 - 18 °C	0°	1.78 ± 0.04	5.58 ± 0.06	0.88 ± 0.10
		45°	1.97 ± 0.03	5.85 ± 0.05	1.16 ± 0.08
		90°	1.90 ± 0.06	5.75 ± 0.04	1.36 ± 0.21
Wet	pH 7.6 - 14 °C	0°	1.42 ± 0.09	5.00 ± 0.03	0.81 ± 0.02
		45°	1.43 ± 0.17	5.02 ± 0.27	0.98 ± 0.13
		90°	1.54 ± 0.07	5.14 ± 0.96	1.31 ± 0.86
	pH 7.6 - 18 °C	0°	1.39 ± 0.07	4.96 ± 0.09	0.80 ± 0.20
		45°	1.37 ± 0.03	4.92 ± 0.05	0.75 ± 0.13
		90°	1.52 ± 0.03	5.03 ± 0.12	1.21 ± 0.07
	pH 8.1 - 14 °C	0°	1.48 ± 0.10	5.08 ± 0.16	0.93 ± 0.15
		45°	1.42 ± 0.11	5.00 ± 0.17	0.73 ± 0.02
		90°	1.39 ± 0.05	4.70 ± 0.08	1.22 ± 0.09
	pH 8.1 - 18 °C	0°	1.51 ± 0.02	5.14 ± 0.02	0.93 ± 0.15
		45°	1.59 ± 0.04	5.26 ± 0.06	0.90 ± 0.08
		90°	1.67 ± 0.04	5.40 ± 0.07	1.28 ± 0.09

Table 2: Geometric dimensions of uniaxial tensile test specimens.
